# Ligustrazine Nanoparticle Hitchhiking on Neutrophils for Enhanced Therapy of Cerebral Ischemia‐Reperfusion Injury

**DOI:** 10.1002/advs.202301348

**Published:** 2023-04-20

**Authors:** Qingchun Mu, Kai Yao, Madiha Zahra Syeda, Min Zhang, Qian Cheng, Yufei Zhang, Rui Sun, Yuting Lu, Huamiao Zhang, Zhicheng Luo, Hanning Huang, Xiaojing Liu, Chunmei Luo, Xiulong Zhu, Shuyu Wu, Liao Cui, Chunming Huang, Xiaoyuan Chen, Longguang Tang

**Affiliations:** ^1^ The People's Hospital of Gaozhou Guangdong Medical University Maoming 525200 China; ^2^ Department of Neurosurgery First Affiliated Hospital of Harbin Medical University Harbin 150001 China; ^3^ International Institutes of Medicine The Fourth Affiliated Hospital Zhejiang University School of Medicine Yiwu 322000 China; ^4^ Basic Medical College Guilin Medical University Guilin 541199 China; ^5^ School of Pharmaceutical Sciences Guangdong Provincial Key Laboratory of New Drug Screening Southern Medical University Guangzhou 510515 China; ^6^ Department of Neurosurgery Hainan General Hospical Hainan Affiliated Hospital of Hainan Medical University Haikou 570311 China; ^7^ Guangdong Provincial Key Laboratory of Research and Development of Natural Drugs and School of Pharmacy Guangdong Medical University Dongguan 523808 China; ^8^ Departments of Diagnostic Radiology and Surgery Clinical Imaging Research Centre Centre for Translational Medicine Nanomedicine Translational Research Program NUS Center for Nanomedicine Yong Loo Lin School of Medicine Departments of Chemical and Biomolecular Engineering and Biomedical Engineering Faculty of Engineering National University of Singapore Singapore 117597 Singapore

**Keywords:** cerebral ischemia‐reperfusion injury, drug delivery, ligustrazine, neutrophils, reactive oxygen species (ROS)

## Abstract

Ischemic stroke is a refractory disease that endangers human health and safety owing to cerebral ischemia. Brain ischemia induces a series of inflammatory reactions. Neutrophils migrate from the circulatory system to the site of cerebral ischemia and accumulate in large numbers at the site of inflammation across the blood–brain barrier. Therefore, hitchhiking on neutrophils to deliver drugs to ischemic brain sites could be an optimal strategy. Since the surface of neutrophils has a formyl peptide receptor (FPR), this work modifies a nanoplatform surface by the peptide cinnamyl‐F‐(D)L‐F‐(D)L‐F (CFLFLF), which can specifically bind to the FPR receptor. After intravenous injection, the fabricated nanoparticles effectively adhered to the surface of neutrophils in peripheral blood mediated by FPR, thereby hitchhiking with neutrophils to achieve higher accumulation at the inflammatory site of cerebral ischemia. In addition, the nanoparticle shell is composed of a polymer with reactive oxygen species (ROS)‐responsive bond breaking and is encased in ligustrazine, a natural product with neuroprotective properties. In conclusion, the strategy of hitching the delivered drugs to neutrophils in this study could improve drug enrichment in the brain, thereby providing a general delivery platform for ischemic stroke or other inflammation‐related diseases.

## Graphical abstract

Ischemic stroke is a refractory disease that endangers human health and safety owing to cerebral ischemia, which induces a series of inflammatory reactions. In this study, a strategy of hitching the delivered drugs to neutrophils is developed to improve drug enrichment in the brain, thereby providing a general delivery platform for ischemic stroke or other inflammation‐related diseases.

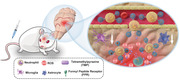

## 1 Introduction

Stroke is associated with a high rate of disability, resulting in permanent disability in ≈50% of survivors, and is one of the leading cause of death and disability among adults worldwide.^[^ ^1^ ^]^ It is mainly classified into hemorrhagic and ischemic stroke. Ischemic stroke is caused by vascular obstruction, usually due to the presence of a thrombus, which accounts for ≈85% of all stroke events.^[^ ^2^ ^]^ Intravenous injection of thrombolytic drugs, such as recombinant tissue plasminogen activator (rtPA), is the only US Food and Drug Administration (FDA)/European Medicines Agency (EMA)‐approved pharmacotherapy for patients with acute ischemic stroke. However, its use is limited by a narrow therapeutic window, selective efficacy, and hemorrhagic complications.^[^ ^3^ ^]^

Ischemic stroke injuries include ischemic and reperfusion injuries. The mechanism of cell death in the ischemic brain involves at least three major processes: ion imbalance due to excitatory toxicity, oxidative/nitrosation stress, and inflammation.^[^ ^4^ ^]^ A series of neuroinflammatory reactions occur, including rapid microglial activation, secretion of inflammatory mediators, and invasion of leukocytes following cerebrovascular occlusion and reperfusion.^[^ ^5^ ^]^ During these processes, neutrophils are usually the first and most abundant inflammatory cells in the microvascular response to ischemic stroke, especially when compared to monocytes/macrophages, which migrate to the ischemic brain region throughout the acute phase of inflammation. An abundance of infiltrative neutrophils is associated with elevated reactive oxygen species (ROS) and inflammatory mediators that exacerbate ischemic tissue damage and contribute to the destruction of the blood‐brain barrier (BBB), cerebral edema, and brain injury.^[^ ^6^ ^]^ This is mediated by factors released by neutrophils, including ROS, proteases, and cytokines.^[^ ^7^ ^]^ ROS are produced shortly after vascular occlusion clearance as well as in the late phase of ischemia‐reperfusion and are major mediators of reperfusion injury.^[^ ^8^ ^]^

Ligustrazine, also called tetramethylpyrazine (TMP), is a bioactive ingredient derived from the traditional Chinese herbal medicine Rhizoma Ligustici Chuanxiong, and has been proven to be effective in protecting neurons and inhibiting inflammation after ischemic brain injury.^[^ ^9^ ^]^ However, TMP has the disadvantages such as a short half‐life, poor water solubility, and low bioavailability.^[^ ^10^ ^]^ To improve the bioavailability of TMP in vivo and enhance its targeting ability to cerebral ischemia, the key is to design a new drug‐delivery system using modern pharmaceutical preparation technology. Nanoparticles (NPs) can address some of these challenges because they prolong the life of medicines in vital systems, are suitable carriers of insoluble medicines, and can induce neuroprotective effects through the BBB by reducing inflammatory responses.^[^ ^11^ ^]^ Neutrophil‐derived cell membranes and neutrophils have been designed as medical delivery vehicles to enhance the targeting of drug molecules to inflammatory sites. However, the large‐scale production of neutrophil cell membranes is difficult and expensive. Therefore, hitchhiking on peripheral circulation neutrophils in vivo to deliver drugs may be a better option using targeted molecules to bind neutrophils.

Neutrophils have a formyl peptide receptor (FPR) on their surface; therefore, we designed a nanocarrier that targets peptides specifically binding to FPR, cinnamyl‐F‐(D)L‐F‐(D)L‐F (CFLFLF).^[^ ^12^ ^]^ We then intravenously administered these nanoparticles and evaluated their ability to bind to neutrophils in the bloodstream and target sites of inflammation. In addition, the shell of nanoparticles comprised a ROS‐responsive polymer capable of breaking bonds and was loaded with ligustrazine. We further assessed the targeted delivery and release of ligustrazine. Through this novel drug nano carrier‐based drug delivery system, we aimed to enrich the ligustrazine delivered to the ischemic site and achieve precise targeted therapy for cerebral ischemia‐reperfusion injury.

## 2 Results

### 2.1 Rational Design and In Situ Synthesis of Nano‐Medicines Targeting Neutrophils

**Figure 1:**
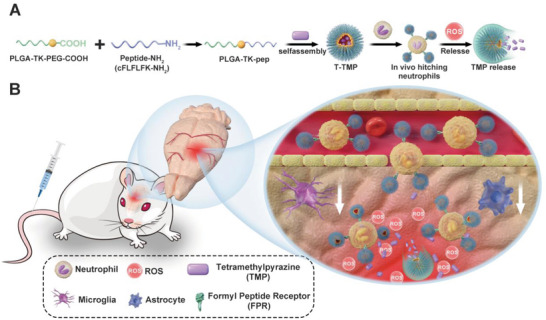
Schematic diagram of preparation of neutrophil targeting and ROS‐responsive ligustrazine nanoparticles (T‐TMP) and its neuroprotective mechanism on cerebral ischemia‐reperfusion injury in middle cerebral artery occlusion (MCAO) model mice. A) T‐TMP was synthesized by self‐assembling with ligustrazine and a formyl peptide receptor (FPR) targeting peptide modified ROS responsive polymer PLGA‐TK‐PEG‐pep. B) T‐TMP injected via the tail vein leads to nanoparticle enrichment by hitching neutrophil migration to cerebral ischemia sites with high ROS expression. The sustained release of TMP from the nanoparticles inhibited the activation of microglia and astrocytes activation and secretion of inflammatory factors, resulting in long‐term therapeutic effects in MCAO mice.

In this study, we designed a type of ROS‐responsive coated nanoparticle mainly composed of poly (lactic*‐co‐*glycolic acid) (PLGA) and polyethylene glycol (PEG). A novel polymer (PLGA‐TK‐PEG) with hydrophilic and hydrophobic properties was formed by synthesizing a ROS‐cleavable ketal (TK) as the intermediate chain connecting PLGA and PEG. The carboxyl‐terminal of PEG was connected to the peptide cinnamyl‐F‐(D) L‐F‐(D) L‐F (CFLFLF), which could target the neutrophil formyl peptide receptor. Finally, the peptide modified polymer (PLGA‐TK‐pep) was used as a ROS‐sensitive and neutrophil targeting nanomaterial to encapsulate the therapeutic drug ligustrazine, denoted as T‐TMP (**Figure** **1**A); Subsequently, by intravenous administration, the nanomedicine T‐TMP was quickly transported by neutrophils to the cerebral ischemic site, where the inflammatory ROS were abundantly expressed. ROS destroyed and cleaved the intermediate TK bond, eventually destroying the nanoparticles and rapidly releasing the encapsulated TMP from the nanoparticles. Finally, drugs were released in a real‐time, targeted manner to the cerebral ischemia‐reperfusion injury site, and the increase in ROS, level of microglia, astrocyte‐induced neuroinflammation, and other adverse reactions caused by cerebral ischemia‐reperfusion injury were reduced (Figure 1B).

### 2.2 Preparation and Characterization of NT‐TMP and T‐TMP

**Figure 2:**
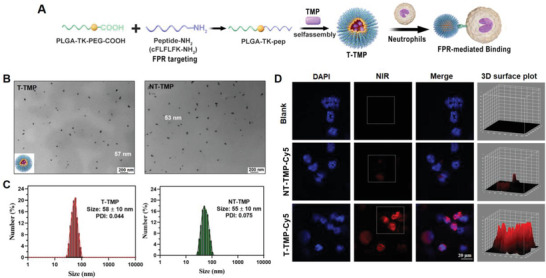
The preparation of T‐TMP nanoparticles that can specifically bind to neutrophils in vitro. A) Schematic diagram of the preparation of T‐TMP and hitching neutrophils through FPR receptor. B,C) Representative TEM (B) and DLS images (C) of T‐TMP and NT‐TMP nanoparticles. D) Confocal images of activated neutrophils for the uptake of Cy5‐labeled T‐TMP and NT‐TMP in vitro.

The preparation process of T‐TMP nanoparticles is illustrated in **Figure** **2**A. For comparison, NT‐TMP was prepared by self‐assembly of PLGA‐TK‐PEG‐mPEG and TMP. The average sizes of T‐TMP and NT‐TMP, as measured by transmission electron microscopy (TEM), were 57 and 53 nm, respectively (Figure 2B). The average hydrodynamic diameters were determined using dynamic light scattering (DLS) (Figure 2C). The zeta potentials of NT‐TMP and T‐TMP detected by DLS were negative (Figure S1A, Supporting Information). The ROS‐responsive drug‐release behavior of T‐TMP was evaluated by incubation with or without H_2_O_2_. The results illustrated that the release rate of TMP in the H_2_O_2_‐treated group was much higher and faster than that in the non‐treated group (Figure S1B, Supporting Information). The T‐TMP NPs exhibited optimal stability after a week of storage at 4 °C (Figure S1C, Supporting Information). Activated neutrophils were incubated with indocyanine green (Cy5)‐labeled NPs and visualized under a confocal microscope. We observed that compared to that in the NT‐TMP and blank groups, neutrophils in the T‐TMP treated group exhibited stronger red fluorescence, indicating that T‐TMP exhibited higher uptake by neutrophils (Figure 2D). Flowcytometric analysis further confirmed the uptake of nanoparticles by neutrophils (Figure S2, Supporting Information). Surface binding of nanoparticles on neutrophils was also clearly observed in our confocal microscopy images (Figure S3, Supporting Information). Subsequently, Cy5‐labeled NPs phagocytosed by neutrophils were monitored by flow cytometry analysis, and the internalization assessment results were consistent with those of confocal microscopy (Figure S4, Supporting Information). Dichlorodihydrofluorescein diacetate (DCFH‐DA) assay was used to evaluate the ROS clearance of drugs in primary astrocytes under H_2_O_2_ stimulation, which revealed that T‐TMP could effectively clear ROS in cells (Figure S5, Supporting Information).

### 2.3 Evaluation of Neutrophil and Inflammation Targeting Ability of T‐TMP in Cerebral Ischemia by NIR‐II Imaging

**Figure 3:**
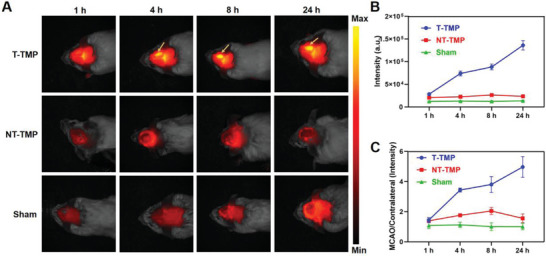
Near infrared two‐region (NIR‐II) imaging results of T‐TMP‐ICG and NT‐TMP‐ICG in MCAO model. Brain NIR‐II imaging was performed after MCAO model mice were intravenously injected with NT‐TMP‐ICG and T‐TMP‐ICG. A) Representative NIR‐II images of mice in the Sham, NT‐TMP‐ICG, and T‐TMP‐ICG groups were recorded at the designated time points after i.v. injection (given immediately after the surgery), which showed the significant accumulation of T‐TMP‐ICG in ischemia area. B) The quantity analysis of NIR‐II signal intensity of the brain ischemia area in each group at different time points. C) NIR‐II signal intensity ratio of MCAO/Contralateral hemispheres of mice in each group (*n* = 3 per group).

To further improve the imaging quality of near‐infrared fluorescence imaging technology, near‐infrared II (NIR‐II, 1000–1700 nm) biomedical fluorescence imaging has recently been developed, which provides a highly versatile platform for non‐invasive in vivo biological imaging that allows for deeper and clearer detection of biological tissues or organs.^[^ ^13^ ^]^ We evaluated the in vivo targeting of T‐TMP and NT‐TMP to ischemic areas of the brain tissue by NIR‐II imaging. The nanoparticles were synthesized by encapsulating ICG, a near‐infrared dye, and were administered intravenously to middle cerebral artery occlusion (MCAO) mice. In vivo imaging of the brain tissue was performed at 1, 4, 8, and 24 h post‐injection to investigate whether T‐TMP could be more efficiently delivered to the cerebral ischemic sites in MCAO mice. The accumulation of fluorescence intensity in the ischemic area of the T‐TMP group was the highest, and no T‐TMP was allowed to enter the brain tissue of the sham group (**Figure** **3**A). Quantitative analysis revealed that 1 h after injection, compared to that in the NT‐TMP and sham groups, the fluorescence signal intensity of NIR‐II in the ischemic brain tissue of the T‐TMP group was significantly increased and reached the highest point at 24 h (Figure 3B). The fluorescence signal intensity of the ischemic brain tissue/contralateral brain tissue in the T‐TMP group was significantly higher than that in the NT‐TMP and sham groups (Figure 3C), which was consistent with the results shown in Figure 3A. These results collectively indicated that T‐TMP was more likely to enter ischemic brain tissue through the blood–brain barrier, and T‐TMP exhibited a higher brain‐targeting efficiency. Moreover, we also performed the ex vivo NIR‐II imaging of other major organs and tested the body toxicity by measuring the parameters of liver (ALT, AST) and kidney function (CK‐MB, CREA) (Figure S6, Supporting Information). In addition, we performed the blood hemolysis experiment to confirm the effect of nanoparticles on the blood cells hemolysis. (Figure S7, Supporting Information). Altogether, these results indicated the general safety of nanoparticles in this study.

**Figure 4:**
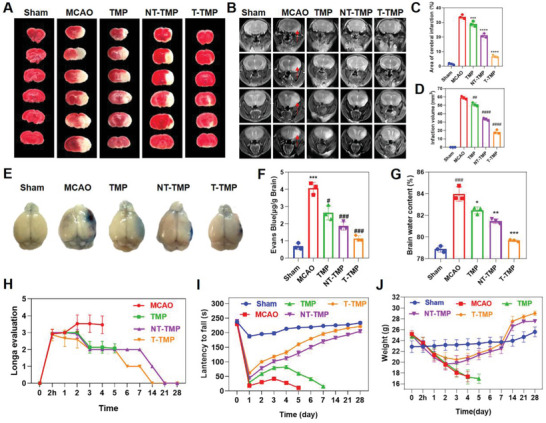
T‐TMP reduces brain tissue infarction, brain edema, penetrability of Evans Blue (EB), and neurological deficits induced by MCAO. A) T‐TMP significantly reduced the brain infarction evaluated by TTC staining at the third day after the mouse MCAO. B) Brain edema was evaluated by magnetic resonance T2‐weighted imaging which showed that the T‐TMP significantly reduced the size of high‐signal area in the brain tissue on T2 image and the infarct volume in brain tissue. C,D) Quantitative analysis of cerebral infarction percentage calculated from TTC (C), and cerebral infarction volume calculated from MRI (D). E) EB staining was used to detect the degree of damage to the blood brain barrier (BBB). T‐TMP notably reduced the extravasation of EB in brain tissue. F) Quantitative analysis of the concentration of EB in the brain tissue. G) The amount of brain edema was calculated from the ratio of dry to wet weights at 72 h after MCAO, which showed that T‐TMP remarkably reduced the water content of brain tissue. H,I) After mouse MCAO, the neurological recovery of mice in each group at different time points was evaluated by Longa score (H) and rotarod test (I). J) Monitoring the effects of TMP, NT‐TMP, and T‐TMP on the body weight of mice at different time points. ***p < 0.001 versus Sham; #*p* < 0.05 versus MCAO. ##*p* < 0.01 versus MCAO, ###*p* < 0.001 versus MCAO, ####*p* < 0.0001 versus MCAO.

T‐TMP reduced infarcts in brain tissue, cerebral edema, permeability of Evans Blue (EB), and neurological deficits caused by MCAO. To further confirm the protective effect of T‐TMP on the brains of mice, we established an MCAO mouse model to simulate the process of cerebral stroke ischemia‐reperfusion. In the MCAO model, a filament with a silica gel tip was inserted into the middle cerebral artery of the mouse for 90 min. Subsequently, the filament was removed to induce reperfusion injury. The effects of the different drugs on cerebral protection in mice were also evaluated. As shown in **Figure** **4**A, large‐scale infarction occurred in the moue MCAO model compared to that in the sham group 3 days after MCAO (*p* < 0.001), which was characterized by the staining with 2,3,5‐triazole chloride (TTC). TMP, NT‐TMP, and T‐TMP reduced the cerebral infarct area (*p* < 0.01, *p* < 0.0001, and *p* < 0.0001, respectively). The reduction in the cerebral infarct area of MCAO mice was the most significant after T‐TMP injection (Figure 4C). Delivery of the T‐TMP to brain was confirmed via immunofluorescent staining of the brain sections and analyzing the inflammatory regions, which showed presence of the nanoparticles bound neutrophils (Figure S8, Supporting Information). On consecutive coronary T2‐weighted magnetic resonance imaging (MRI) scans, the cerebral infarct lesion showed a hyperintense area in the right brain. The infarct size was significant in the MCAO group and decreased significantly on day 3 after MCAO following treatment with TMP, NT‐TMP, and T‐TMP (*p* < 0.01, *p* < 0.0001, and *p* < 0.0001, respectively) (Figure 4B,D). Studies have shown an increased National Institutes of Health Stroke Scale (NIHSS) score, functional outcome, and mortality in patients with ischemic stroke with severe blood–brain barrier damage compared to those in patients with mild blood–brain barrier damage. Therefore, improving the blood–brain barrier damage can improve the neurological prognosis of patients with stroke.^[^ ^14^ ^]^ Thus, we studied the permeability of the blood–brain barrier by measuring the extravasation of Evans Blue. We observed a greater amount of Evans Blue dye extravasation in the cortex of the MCAO group 3 days after MCAO than in the sham group (*p* < 0.001). Compared to that in the MCAO group, extravasation of Evans Blue dye was significantly reduced in the TMP, NT‐TMP, and T‐TMP groups (*p* < 0.01, *p* < 0.0001, and *p* < 0.0001, respectively) (Figure 4E,F). 3 days after MCAO, we measured the brain water content of the mice in each group using the dry‐wet weight method. We observed an increase in the percentage of brain water content in the injured hemisphere 3 days after MCAO compared to that in the sham group (*p* < 0.001). Brain water content was significantly reduced (*p* < 0.01, *p* < 0.0001, and *p* < 0.0001, respectively) with treatment with TMP, NT‐TMP, and T‐TMP (Figure 4G). After treatment with TMP, NT‐TMP, and T‐TMP, we analyzed the Logna score, and the rotarod assay was performed to evaluate the effects of nanoparticles on neurological function and behavior in MCAO mice. Starting from the second day after MCAO, the Logna scores of MCAO mice treated with TMP, NT‐TMP, and T‐TMP were significantly lower than those of mice in the MCAO group, and neurological function was significantly improved. 5 days after MCAO, all MCAO group mice died. Neurological function was the most significantly improved in the T‐TMP treatment group (Figure 4H). The results of the rotarod test revealed that from the first day after MCAO, the falling time of the mice in the TMP, NT‐TMP, and T‐TMP groups from the rotarod was longer than that of the mice in the MCAO group. The falling time of the mice in the T‐TMP group from the rotarod was the longest (Figure 4I). These results suggested that neurological function most significantly improved in the T‐TMP treatment group. Mice in the MCAO and TMP groups died on day 6 and 8, respectively. The body weights of MCAO mice began to decrease on the first day after MCAO, and all mice died on the fifth day after MCAO. The body weights of NT‐TMP‐ and T‐TMP‐treated MCAO mice began to increase on the fourth day after MCAO (Figure 4J). HE staining results indicated that none of the medicines were toxic to the tissues and organs of mice (Figure S9, Supporting Information).

### 2.4 Inhibition of T‐TMP on Inflammation and Immune Response Induced by Ischemic Stroke Reperfusion

**Figure 5:**
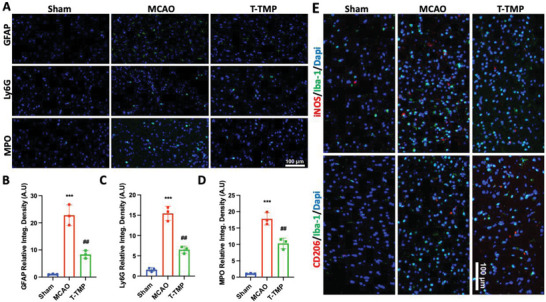
T‐TMP can inhibit the progression of neuroinflammation in MCAO mice. A) Immunofluorescence assays confirmed the expression of microglia, astrocytes, neutrophils, and myeloperoxidase (MPO) 3 days after MCAO in different experimental groups. B–D) Quantitative analysis showed that T‐TMP could significantly reduce astrocyte (B), and neutrophil (C) infiltration and MPO (D) production in the brain tissue after MCAO. E) Representative immunofluorescent images of M1 (iNOS/Iba‐1/DAPI)) and M2 microglia (CD206/Iba‐1/DAPI) show decrease in M1 and increase in M2 microglia in the T‐TMP group compared to the MCAO model. ***p < 0.001 versus Sham, ##*p* < 0.01 versus MCAO.

In ischemic brain tissue, astrocytes exhibited strong hypertrophy and proliferation changes in the ischemic penumbra. Microglia showed coarse process branch cells or fine process deformation cells, indicating that these two types of glial cells were activated after ischemic injury.^[^ ^15^ ^]^ In cerebral ischemic brain tissue, activated microglia can produce pro‐inflammatory cytokines, such as interleukin‐1*β* (IL‐1*β*), interleukin‐6 (IL‐6), and tumor necrosis factor *α* (TNF‐*α*).^[^ ^16^ ^]^ Although the primary purpose of activating microglia is to protect neuronal cells, microglial overactivation can lead to harmful inflammation and neuronal death.^[^ ^17^ ^]^ The activation is extensive and persistent during cerebral ischemia. The activation of NF‐*κ*B induces inflammasome formation and astrocyte release of IL‐1*β*.^[^ ^18^ ^]^ Compared with the sham operation group, immunofluorescence staining of the mouse brain tissue 3 days after MCAO showed significantly increased microglia, astrocytes, neutrophils, and myeloperoxidase (MPO) (*p* < 0.001) (**Figure** **5**A). Treatment with T‐TMP significantly reduced the number of astrocytes (Figure 5B), and neutrophils (*p* < 0.01 and *p* < 0.01, respectively) (Figure 5C) and reduced the expression of MPO (*p* < 0.01) (Figure 5D). Considering the negative role of M1 microglia in brain damage, we performed immunofluorescent staining of brain sections to detect the expression of M1 and M2 microglia after T‐TMP treatment. Results show a decrease in M1and increase in M2 microglia in the T‐TMP treated group compared to the MCAO model (Figure 5E). We also performed immunofluorescent staining of whole brain sections to test the distribution of T‐TMP in the inflammatory and non‐inflammatory regions. Results showed an enrichment of nanoparticles in the inflammatory region compared to the non‐inflammatory region of brain tissue. (Figure S10, Supporting Information). Altogether, these results demonstrate that nanoparticles can effectively target the injury site, and that T‐TMP treatment can improve the post‐ischemic inflammatory response by reducing the levels of pro‐inflammatory cytokines.

### 2.5 T‐TMP Reduced ROS and Apoptosis Induced by MCAO

**Figure 6:**
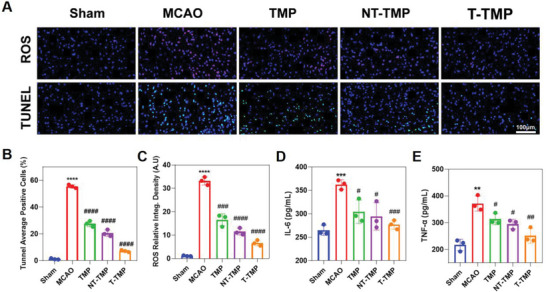
Treatment with T‐TMP reduced the expression of ROS, apoptosis, and the production of inflammatory factors in the brain tissue of MCAO in mice. A) Immunofluorescence assays confirmed the expression of ROS and apoptosis 3 days after MCAO in different experimental groups. B–E) T‐TMP significantly reduced ROS expression (B), apoptosis (C), and inhibited the production of IL‐6 (D) and TNF‐*α* (E). **p < 0.01 versus Sham, ***p < 0.001 versus Sham, ****p < 0.0001 versus Sham; #*p* < 0.05 versus MCAO, ##*p* < 0.01 versus MCAO, ###*p* < 0.001 versus MCAO, ####*p* < 0.0001 versus MCAO.

In addition, TdT‐mediated dUTP nick‐end labeling (TUNEL) assay results demonstrated that brain tissues exhibited typical apoptotic characteristics on day 3 after MCAO compared to that in the sham group (*p* < 0.0001). TMP, NT‐TMP, and T‐TMP treatments significantly reduced apoptosis (*p* < 0.0001, *p* < 0.0001, and *p* < 0.0001, respectively) (**Figure** **6**A,B). ROS were significantly expressed in the brain tissue on day 3 after MCAO compared to that in the sham group (*p* < 0.0001) (Figure 6A). TMP, NT‐TMP, and T‐TMP significantly reduced ROS expression in the brain tissue (*p* < 0.001, *p* < 0.0001, and *p* < 0.0001, respectively) (Figure 6C). The enzyme‐linked immunosorbent assay (ELISA) results showed significantly increased IL‐6 (*p* < 0.001) and TNF‐*α* (*p* < 0.01) protein levels in the MCAO group 3 days after MCAO compared to those in the sham group. The TMP, NT‐TMP, and T‐TMP groups had significantly lower levels of IL‐6 (*p* < 0.05, *p* < 0.05, *p* < 0.001, respectively) (Figure 6D) and TNF‐*α* (*p* < 0.05, *p* < 0.05, *p* < 0.01, respectively) (Figure 6E) than in the MCAO group. These results indicated that T‐TMP inhibited apoptosis more effectively and thus further reduced oxidative damage caused by cerebral stroke ischemia‐reperfusion.

## 3 Discussion

Inflammation is the immune response characterized by a marked increase of immune cells and their trafficking from bloodstream to pathogenic tissues.^[^ ^19^ ^]^ Millions of polymorphonuclear neutrophils, a type of white blood cells, can rapidly respond to inflammation through neutrophil activation, adhesion to and migration across endothelial vessels into inflammatory tissues via the intercellular route.^[^ ^7^, ^20^ ^]^Therefore, neutrophils could be an excellent carrier to mediate the delivery of therapeutic NPs across the endothelial vessel barrier and to specifically target diseased tissues.

Cerebral ischemia‐reperfusion injury has been shown to be strongly associated with acute inflammation. Local administration of drugs is not feasible for some inflammatory diseases, because the drugs lack tissue penetration ability after systemic delivery, and may also cause systemic toxicity. Targeted drug delivery and drug retention in extravascular diseased locations are prerequisites for pharmacological therapies and are also the primary goals of nanomedicine.^[^ ^21^ ^]^ For medical benefit, therapeutic nanoparticles (NPs) should cross the blood‐brain barrier.^[^ ^22^ ^]^ Studies have shown the use of nanotechnology which target neutrophils to deliver the drugs^[^ ^23^ ^]^ to treat lung infection,^[^ ^24^ ^]^ atherosclerosis,^[^ ^25^ ^]^ stroke,^[^ ^21^, ^26^ ^]^ and even cancer.^[^ ^27^ ^]^ Neutrophils act as the initial responders and quickly migrate to the site of ischemic injury. Thus offering a unique opportunity to deliver drugs across the blood brain barrier. Therefore neutrophils could be the excellent carriers to mediate delivery of therapeutic nanoparticles across the blood brain barrier and specifically target the ischemic site. Here, we developed a novel and multifunctional ligustrazine nanoparticle platform for cerebral ischemia‐reperfusion injury therapy by hitchhiking on neutrophils. There is a possibility of other FPR expressing leukocytes^[^ ^28^ ^]^ including monocytes and macrophages to uptake the nanoparticles as well. However, while other leukocytes also arrive at the ischemic site in substantially lesser numbers, neutrophils are the initial responders to arrive at the ischemic site and peak at 72 h.^[^ ^29^ ^]^ Thus, in this study we focused on the neutrophils.

## 4 Conclusion

In summary, we have developed a novel and multifunctional ligustrazine nanoparticle platform was developed for cerebral ischemia‐reperfusion injury therapy by hitchhiking on neutrophils. Ligustrazine nanoparticles could effectively bind neutrophils circulating in the blood and rapidly accumulate around ischemic brain injury tissues. Ligustrazine is released in response to ROS stimulation and can inhibit the inflammatory cell infiltration, suppress the expression of inflammatory factors, alleviate neurological loss, and provide neuroprotection. Our results are in line with the other studies that have reported the neutrophil hitchhiking for targeted delivery to the site of injury to alleviate the disease. This novel nanoplatform designed for ligustrazine delivery has increased the drug uptake and retention at the cerebral ischemia site. Further, our nanocarrier provides an effective strategy for targeted delivery and could be used for the spatiotemporally controlled release of other drugs.

## 5 Experimental Section

### Materials

Ligustrazine was purchased from Solarbor (Beijing, China). Other chemicals were purchased from Alfa Aesar, Thermo Fisher Scientific, or Sigma‐Aldrich and used without further purification. Ultrapure water was prepared using a Milli‐Q Plus System. ELISA kits were purchased from Thermo Fisher Scientific. The TUNEL Assay Kit was obtained from Roche Diagnostics Corp.

### Preparation of NT‐TMP and NT‐TMP‐ICG Nanoparticles

For NT‐TMP NPs, a solution of TMP (4 mg) and PLGA‐TK‐mPEG2K (40 mg) in THF (2 mL) was added into ultrapure water (2 mL) dropwise under stirring and kept in a fume hood overnight to evaporate the THF. For NT‐TMP‐ICG NPs, a solution of TMP (2 mg) and PLGA‐TK‐mPEG2K (40 mg) in THF (2 mL) was added into ICG aqueous solution (2 mL, 1 mg mL^−1^) dropwise under stirring and kept in a fume hood overnight to evaporate the THF. Subsequently, the obtained NT‐TMP NPs and NT‐TMP‐ICG NPs were purified using ultrafiltration tubes (MWCO, 3000 Da) and stored at 4 °C for further use.

### Preparation of T‐TMP and T‐TMP‐ICG NPs

For T‐TMP NPs, a solution of TMP (4 mg) and PLGA‐TK‐PEG2K‐COOH (40 mg) in THF (2 mL) was added into ultrapure water (2 mL) dropwise under stirring and kept in a fume hood overnight to evaporate the THF. For T‐TMP‐ICG, a solution of PLGA‐TK‐PEG2K‐COOH (40 mg) in THF (2 mL) was added into ICG aqueous solution (2 mL, 1 mg mL^−1^) dropwise under stirring and kept in a fume hood overnight to evaporate the THF. The carboxy‐T‐TMP NPs and carboxy‐T‐TMP‐ICG NPs were afterward incubated with EDC (10 mg mL^−1^) in MES buffer (2 mL,10 mm, pH 6.0) for 30 min under gentle stirring and kept stirring for an additional 3 h after formyl peptide receptor (FPR) targeting peptide cinnamyl‐F‐(D)L‐F‐(D)L‐F (CFLFLF) (15 mg) was added. The obtained T‐TMP and T‐TMP‐ICG were purified via an ultrafiltration tube (MWCO, 3000 Da) and stored at 4 °C for the following use.

### Animal Model

The animal experiments were performed following a protocol approved by the Animal Care and Use Committee of Zhejiang University (approval number: ZJU20220247). Adult male C57BL/6 mice weighing 20–25 g and aged 6–8 weeks were provided by the Animal Center of Zhejiang University (Zhejiang, China) and maintained under a 12‐h light/dark cycle with free access to water and food. Briefly, mice were anesthetized with 2% isoflurane. After making a midline incision in the neck, the right common carotid, external and internal carotid arteries were dissected. A 6‐0 silica‐coated nylon monofilament was inserted into the external carotid artery and advanced along the internal carotid artery and occluded the origin of the middle cerebral artery. After 90 min of occlusion, the monofilament was removed to allow for reperfusion. The incision was sutured, and mice were allowed to recover when they were kept warm under a heating lamp. Mice were weighed at different time points after MCAO. Mice were randomly assigned to the following experimental groups before surgery: Sham, MCAO, TMP, NT‐TMP, and T‐TMP. First, Ligustrazine was dissolved in DMSO and then diluted in PBS to a final concentration of 4 mg mL^−1^. Free TMP or TMP nanoparticles (T‐TMP, NT‐TMP) (32 mg kg^−1^ of TMP) were administered via the tail vein immediately, on day 2 and 4 after MCAO. The sham group was established, consistent with the treatment of MCAO group except for the occlusion. The MCAO group injected an equal volume of PBS compared with other experimental groups.

### NIR‐II Imaging

Sham group mice were injected with T‐TMP‐ICG via the tail vein, and the MCAO mice were similarly injected with NT‐TMP‐ICG and T‐TMP‐ICG. 1, 4, 8, and 24 h after administration, the brain tissue was exposed to NIR‐II Kaer Imaging System (KIS NIR‐II, KAER LABS, France) (808 nm, 50 mW cm^−2^) for 5 min (*n* = 3 per group). Time‐dependent NIR‐II fluorescence images of brain tissue were gained by using in vivo imaging system. The excitation was induced by an 808 nm laser and was filtered with an 808 nm long‐pass filter. An 1100 nm bandpass filter was used to collect fluorescence emission and the exposure time was 50 ms (*n* = 3 per group).

### Longa Test and Rotarod Test

Neurobehavioral dysfunction due to ischemic stroke was measured by Longa test (*n* = 15 per group). The scales were as follow: 0: normal, no neurological deficit; 1: unable to extend the left forelimb completely, mild neurological deficit; 2: failure to walk in a straight line, moderate neurological deficit; 3: Turn to the right (hemiplegia side), severe neurological deficit; 4 points: inability to walk spontaneously, loss of consciousness. Rotarod test was performed to assess balance and motor coordination (*n* = 15 per group). The mice underwent a 2 day training prior to MCAO, which gradually accelerate from 4 to 20 rpm in 5 min. The mean time of latency to fall was measured when the mice fall off the rod in three trails.

### TTC Staining

The mice were sacrificed on day 3 under deep anesthesia (*n* = 3 per group). Brains were cut into six 2 mm thick slices and incubated in 2% 2,3,5‐triphenyltetrazolium chloride (TTC) (LEAGENE, Beijing, China). Sections in TTC solution were incubated at 37 °C for 20 min. Infarct lesion area was measured using Image J analysis software. Unstained areas of brain sections were defined as infarcts. Infarct size was indirectly measured by subtracting the non‐infarcted area in the infarcted hemisphere from the total area in the non‐infarcted hemisphere. The percentage of hemispheric infarct area was calculated according following equation: infarct volume/total volume of non‐infarcted hemisphere × 100%.

### MRI Imaging

The MCAO mice with different treatment were anaesthetized with isoflurane (*n* = 3 per group). T2‐weighted coronal magnetic resonance imaging of the brain were recorded with 3.0 T MR scanner (Siemens, Trio, Germany) with a 7.0 T CG NOVILA system (Chenguang Med Tech Co, Shanghai, China) on day 3 post reperfusion. Image J was used to monitor the infarct volume from high‐signal areas.

### EB Permeability Assay

The decomposition of the BBB was assessed by the EB (MACKLIN, Shanghai, China) exudation technique. A 4% EB was injected (2.5 mL kg^−1^) via the tail vein on day 3 after MCAO. Animals were transcardially perfused with heparinized saline after 6 h of reperfusion (*n* = 3 per group). Photos of the brain were taken before dividing them into two hemispheres. Each hemisphere was homogenized in 1 mL of 50% trichloroacetic acid and centrifuged at 14 000 rpm for 20 min at 4 °C by Microcentrifuge (Ke Cheng, Suzhou, China). The supernatant was collected and the absorbance was measured at 610 nm using spectrophotometry. The EB content from each brain was calculated.

### Brain Water Content

The brain water content was calculated using the wet‐dry method. Mice were sacrificed and the brain was removed on day 3 after MCAO (*n* = 3 per group). The brain tissue were immediately weighed to obtain the wet weight, and then dried at 100 °C for 2 days to determine the dry weight. Brain water content was calculated as follows: (wet weight‐dry weight)/wet weight × 100%.

### Immunofluorescence Staining, ROS detection, TUNEL Staining, and HE staining

3 days after MCAO, the mice were anesthetized and perfused with PBS, followed by perfusion with 4% paraformaldehyde (*n* = 3 per group). Subsequently, the brains were removed and stored in 4% paraformaldehyde at 4 °C overnight. The brain tissues were then embedded in paraffin wax and cut into 5 µm thick sections on a microtome (Leica, Heidelberg, Germany). The coronal sections were stained with primary antibodies: diluted Rabbit Anti‐Iba‐1, GFAP, Ly6G, and MPO (Servicebio, Wuhan, China) overnight at 4 °C. After washing, the coronal sections were visualized by treatment with a secondary antibody (Servicebio, Wuhan, China) and the nuclei were counterstained with 4′, 6‐diamidino‐2‐phenylindole (DAPI) (Servicebio, Wuhan, China). Quantification of the IF staining results was done via image J. Integrated density was calculated which represents the sum of the pixel values in the immunoflourescent microscopic image. The integrated density of sham group was defined as 1. The relative integrated density was then calculated as : Integrated density of each group/integrated density of the sham group (i.e, 1). Graphpad Prism 8 was then used to plot these results into Histogram.

The coronal sections were also stained with ROS staining solution (Servicebio, Wuhan, China) and incubated at 37 °C for 30 min in dark place. TUNEL reactivity was measured by TUNEL Apoptosis Assay Kit (Servicebio, Wuhan, China). Then they were incubated with DAPI solution at room temperature for 10 min in dark place and observed under a fluorescence microscope (Leica, Heidelberg, Germany). The organs, including lung, liver, spleen, kidney, and heart were frozen‐cut in the coronal sections at a thickness of 5 µm and stained with HE.

### Enzyme‐Linked Immunosorbent Assay for Inflammatory Cytokine Determination

3 days after MCAO, the mice were anesthetized and the brain tissue were homogenized (*n* = 3 per group). After 15 min of centrifugation at 500 × g, the supernatants were collected to measure Tumor necrosis factor‐a (TNF‐a) and Interleukin‐1*β* (IL‐1*β*) (Fine test, Wuhan, China) (*n* = 3 per group) in accordance with the manufacturer's protocol.

### Cellular Uptake

The uptake of T‐TMP‐Cy5 and NT‐TMP‐Cy5 by neutrophils was evaluated by confocal laser scanning microscopy (CLSM) and flow cytometry. Neutrophils were seeded in 12‐well plates at a density of 1 × 10^5^ cells per well in 1 mL of fresh medium and cultured for 12 h (*n* = 3 per group). Then, ICG‐labeled T‐TMP (T‐TMP‐Cy5) and NT‐TMP (NT‐TMP‐Cy5) at 2 µg mL^−1^ of Cy5 were added to each well. After coincubation for 24 h, the nuclei were stained with DAPI for CLSM. Cells were collected for quantification by flow cytometry analysis. Neutrophils were seeded in 12‐well plate with a density of 3 × 10^6^ cells per well for 24 h incubation, T‐TMP‐Cy5 and NT‐TMP‐Cy5 solutions at 2 µg mL^−1^ of ICG were added into each well, then the cells were incubated with the solutions for 24 h. Quantification of NPs uptake in neutrophils was measured by flow cytometry (FACSCalibur, Becton Dickinson, USA).

### Measurement of Intracellular ROS

C6 cells were incubated in F12K with FBS (10%), respectively, and penicillin (100 U mL^−1^) and streptomycin (50 U mL^−1^) were added at 37 °C in a 5% CO_2_ incubator (*n* = 3 per group). C6 cells were randomly divided into control group, H_2_O_2_ group, H_2_O_2_+TMP, H_2_O_2_+NT‐TMP, and H_2_O_2_+T‐TMP group (*n* = 3 per group). The cells were incubated with H_2_O_2_ (100 *µ* m) and dugs (50 µm L^−1^) for 1 h, then the C6 cells were stained with 5 µm H2DCFDA (GLPBIO, Nantong, China) for 30 min at 37 °C in the dark. Meanwhile, the Control group did not add drugs, and other experiments were the same as the experimental group. The cells were analyzed for ROS generation using a fluorescence microscope at excitation and emission wavelengths of 488.

### Statistical Analyses

The independent two‐sample *t*‐tests and one‐way analysis of variance (ANOVA) was used for comparison in the sham, PBS, TMP, NT‐TMP, and T‐TMP groups. When the ANOVA test was found to be significant, Tukey's test was used to make pairwise comparisons between groups. GraphPad Prism 8 (GraphPad Software, USA) was used for all statistical analyses. The results are depicted as the mean ± SD, and a *p*‐value < 0.05 was considered to be statistically significant.

## Conflict of Interest

The authors declare no conflict of interest.

## Supporting information

Supporting Information

## Data Availability

The data that support the findings of this study are available from the corresponding author upon reasonable request.
