# Supplementary material for: Ligustrazine Nanoparticle Hitchhiking on Neutrophils for Enhanced Therapy of Cerebral Ischemia‐Reperfusion Injury
Source: Adv Sci (Weinh). 2023 Apr 20;10(19):2301348. doi: 10.1002/advs.202301348 (PMC10323616; doi:10.1002/advs.202301348)
Supplement: Supplementary file 1 — Supporting Information [file ADVS-10-2301348-s001.pdf]

ADVANCED SCIENCE
Open Access

Supporting Information

for *Adv. Sci.*, DOI 10.1002/advs.202301348

Ligustrazine Nanoparticle Hitchhiking on Neutrophils for Enhanced Therapy of Cerebral Ischemia-Reperfusion Injury

Qingchun Mu, Kai Yao, Madiha Zahra Syeda, Min Zhang, Qian Cheng, Yufei Zhang, Rui Sun, Yuting Lu, Huamiao Zhang, Zhicheng Luo, Hanning Huang, Xiaojing Liu, Chunmei Luo, Xiulong Zhu, Shuyu Wu, Liao Cui, Chunming Huang*, Xiaoyuan Chen* and Longguang Tang**

Supporting Information

Ligustrazine nanoparticle hitchhiking on neutrophils for enhanced therapy of cerebral ischemia-reperfusion injury

Qingchun Mu[#,], Kai Yao[#], Madiha Zahra Syeda[#], Min Zhang, Qian Cheng, Yufei Zhang, Rui Sun, Yuting Lu, Huamiao Zhang, Zhicheng Luo, Hanning Huang, Xiaojing Liu, Chunmei Luo, Xiulong Zhu, Shuyu Wu, Liao Cui, Chunming Huang*, Xiaoyuan Chen*, Longguang Tang**

Dr. Q. Mu, Dr. MZ. Syeda, Prof. C. Huang, Dr. Z. Luo, Dr. C. Yang, Dr. H. Huang, X. Liu, C. Luo, Prof. X. Zhu, Prof. L. Tang

The People's Hospital of Gaozhou, Guangdong Medical University, Maoming 525200, China

Email: muq@hainmc.edu.cn; Huangchunming5888@163.com; tanglongguang@zju.edu.cn

Dr. K. Yao

Department of Neurosurgery, First Affiliated Hospital of Harbin Medical University, Harbin 150001, China

M. Zhang, Y. Lu, H. Zhang

International Institutes of Medicine, The Fourth Affiliated Hospital, Zhejiang University School of Medicine, Yiwu 322000, China

Q. Cheng, Prof. Y. Zhang,

Basic Medical College, Guilin Medical University, Guilin 541199, China

R. Sun

School of Pharmaceutical Sciences, Guangdong Provincial Key Laboratory of New Drug Screening, Southern Medical University, Guangzhou 510515, China

Dr. S. Wu,

Department of Neurosurgery, Hainan General Hospical, Hainan Affiliated Hospital of Hainan Medical University, Haikou 570311, China.

Prof. L. Cui,

Guangdong Provincial Key Laboratory of Research and Development of Natural Drugs, and School of Pharmacy, Guangdong Medical University, Dongguan 523808, China

Prof. X. Chen

Departments of Diagnostic Radiology, and Surgery, Clinical Imaging Research Centre, Centre for Translational Medicine, Nanomedicine Translational Research Program, NUS Center for Nanomedicine, Yong Loo Lin School of Medicine; Departments of Chemical and Biomolecular Engineering, and Biomedical Engineering, Faculty of Engineering, National University of Singapore, Singapore 117597, Singapore

Email: chen.shawn@nus.edu.sg

# These authors contributed equally to this work.

Supplementary figures

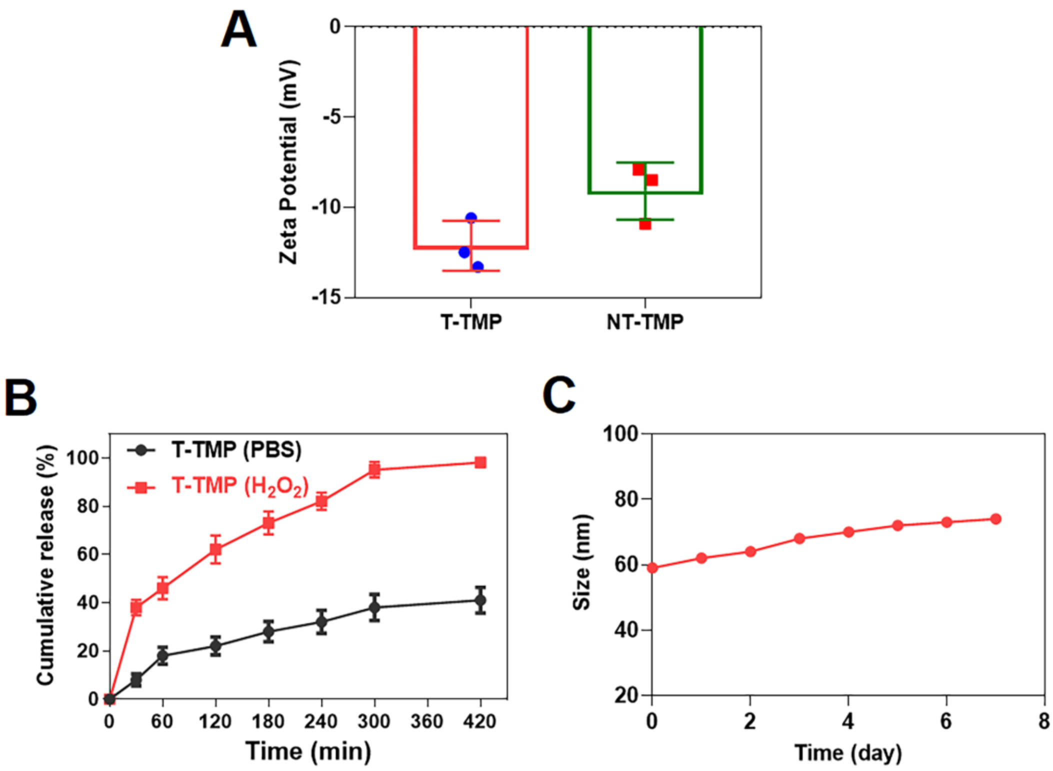

Figure S1. T-TMP Materials Characterization. (A) The zeta potential of NT-TMP and T-TMP nanoparticles. (B) Cumulative release of TMP from NPs in PBS (7.4 pH) and H_2O_2 (1 mM). (C) Stability of T-TMP nanoparticles (NPs) after a week storage in aqueous solution under 4 °C.

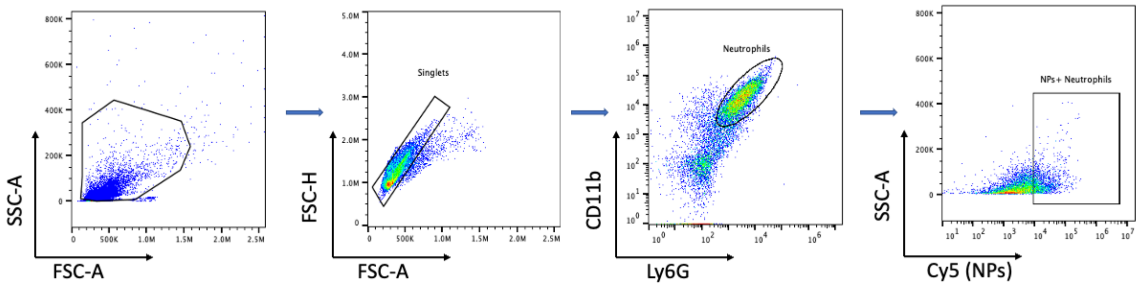

Figure S2. Flowcytometry results of the nanoparticles targeting neutrophils.

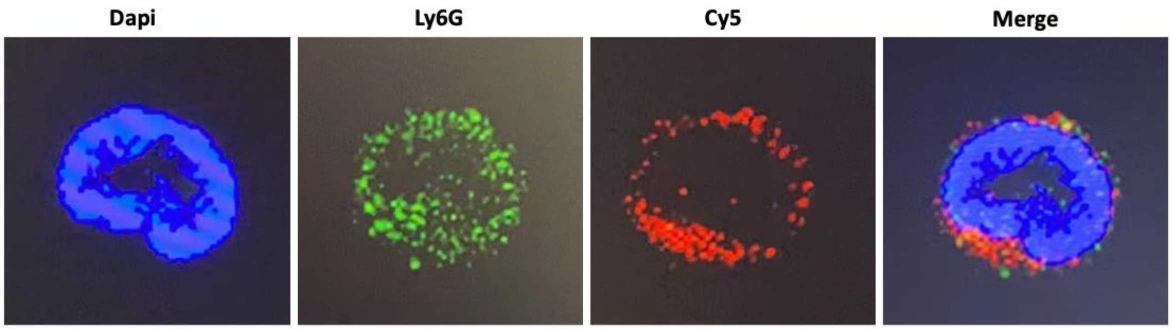

Figure S3. Representative confocal images showing the surface binding of nanoparticles (Cy5) on the surface of neutrophils (Ly6G).

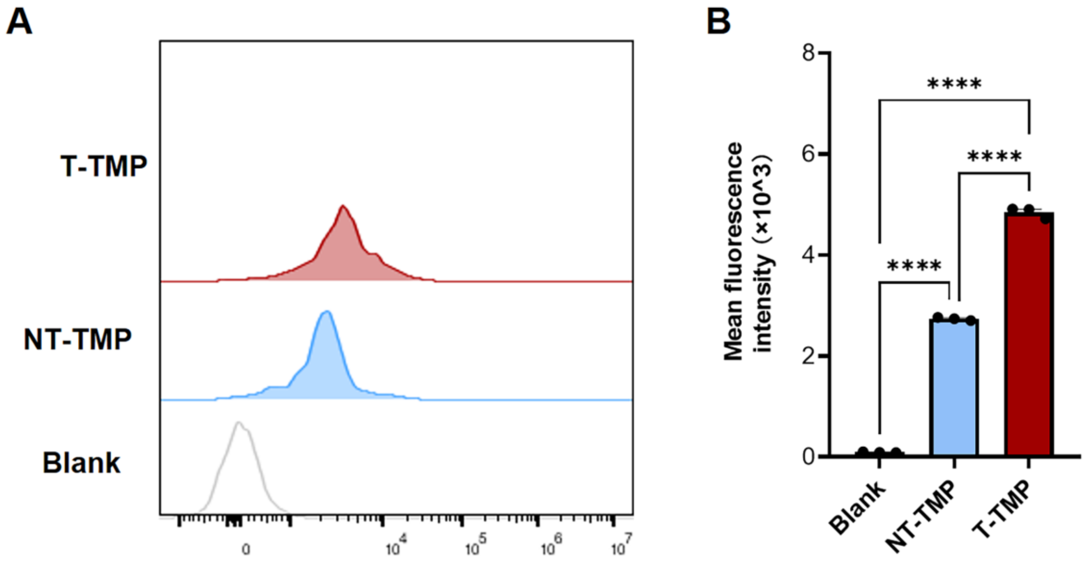

Figure S4. Activated neutrophils uptake Cy5-labeled NT-TMP and T-TMP NPs. (A) The neutrophils uptake Cy5-labeled NPs were performed by flow cytometry analysis.

(B) The quantitative analysis of flow cytometry. ****P <0.0001 (n = 3/group).

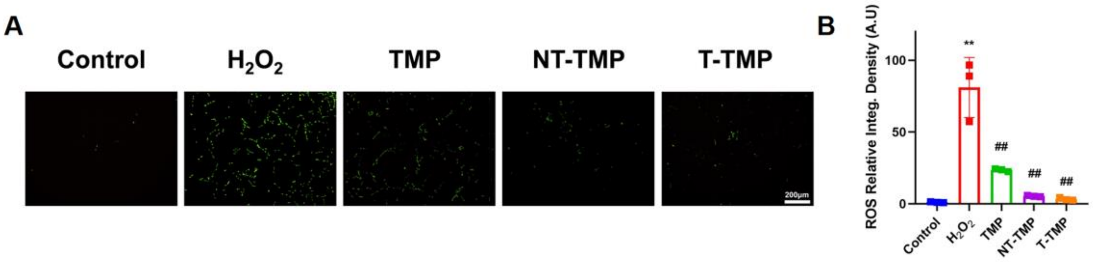

Figure S5. The expression of ROS in C6 cells of different groups was detected by DCFH-DA experiment. (A) Immunofluorescence results showed that T-TMP significantly reduced ROS production in C6 cells induced by hydrogen peroxide. (B) Quantitative analysis of the inhibition of ROS expression in C6 cells by different drugs. **P < 0.01 versus Sham; ##P < 0.01 versus H_2O_2.

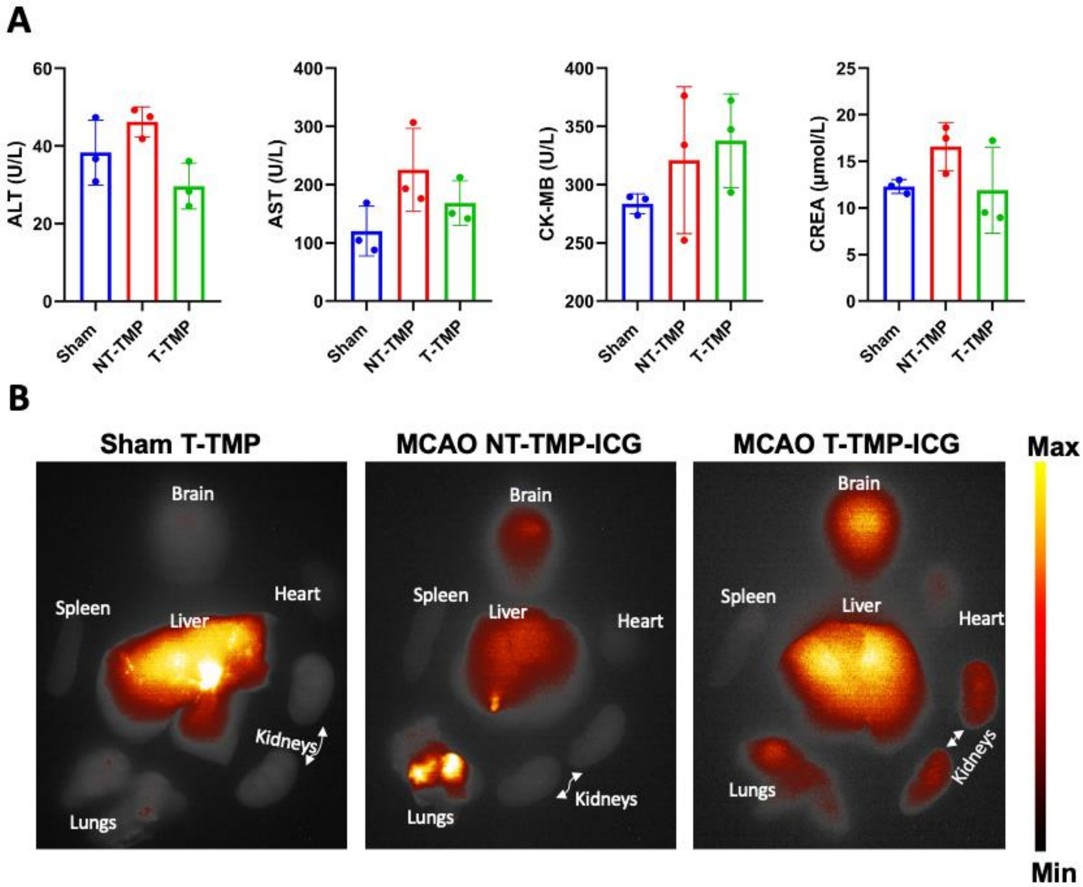

Figure S6. Measurement of **A)** blood toxicity and **B)** biodistribution of nanoparticles *in vivo*.

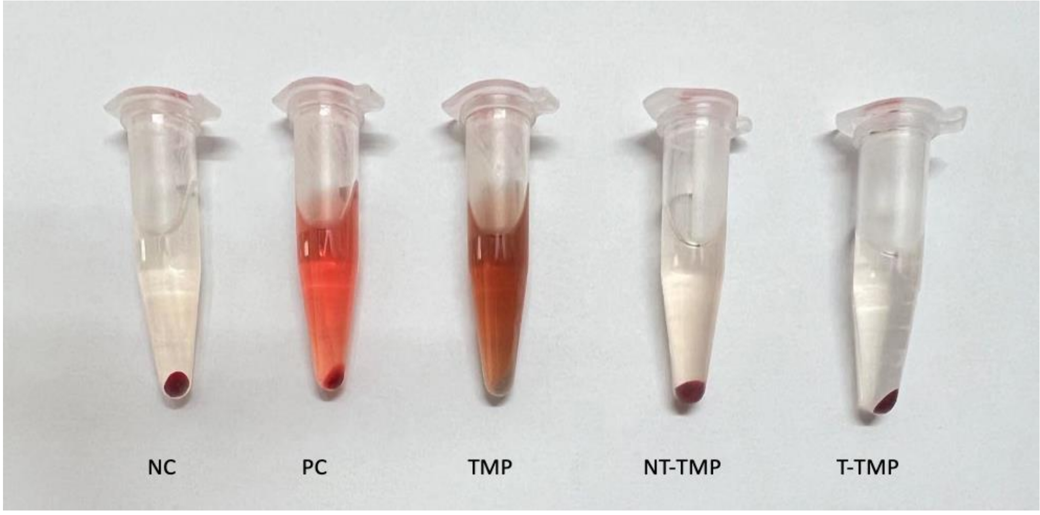

Figure S7. Blood sample was treated with TMP, NT-TMP and T-TMP for 3 hours at room temperature. PBS was used as negative control (NC), and ddH2O was used as positive control (PC).

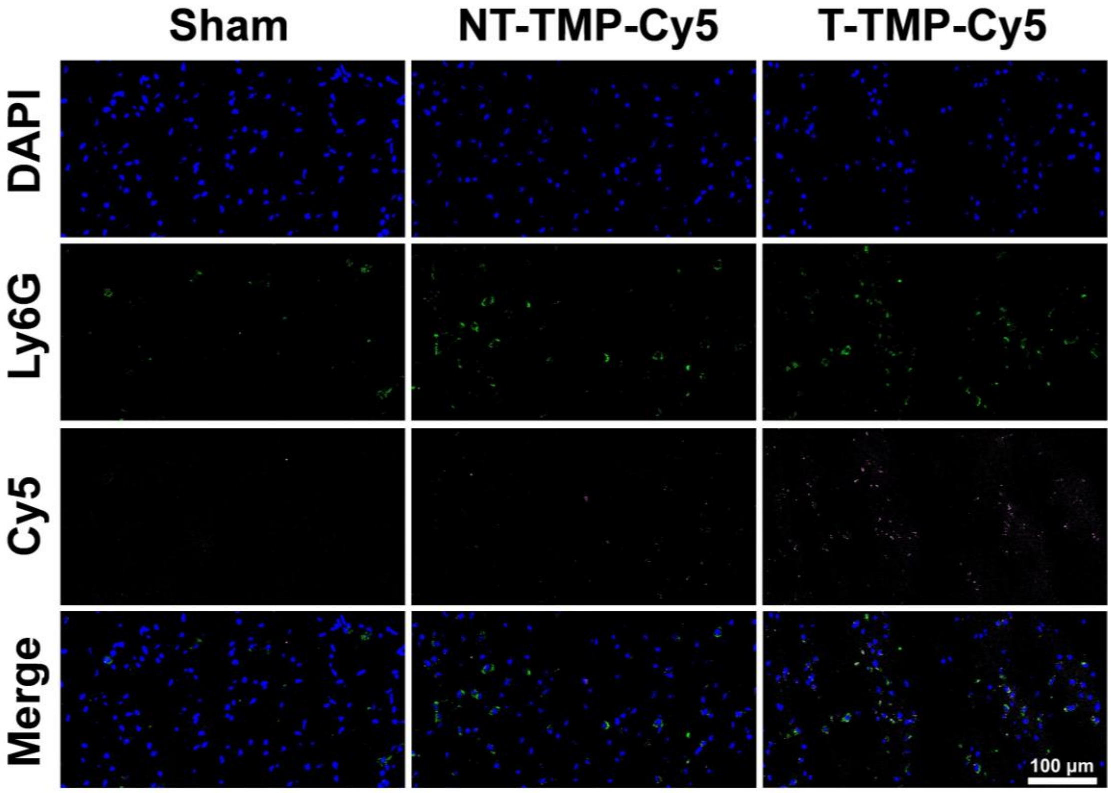

Figure S8. Immunofluorescent staining of brain sections in Sham, NT-TMP-Cy5 and T-TMP-Cy5.

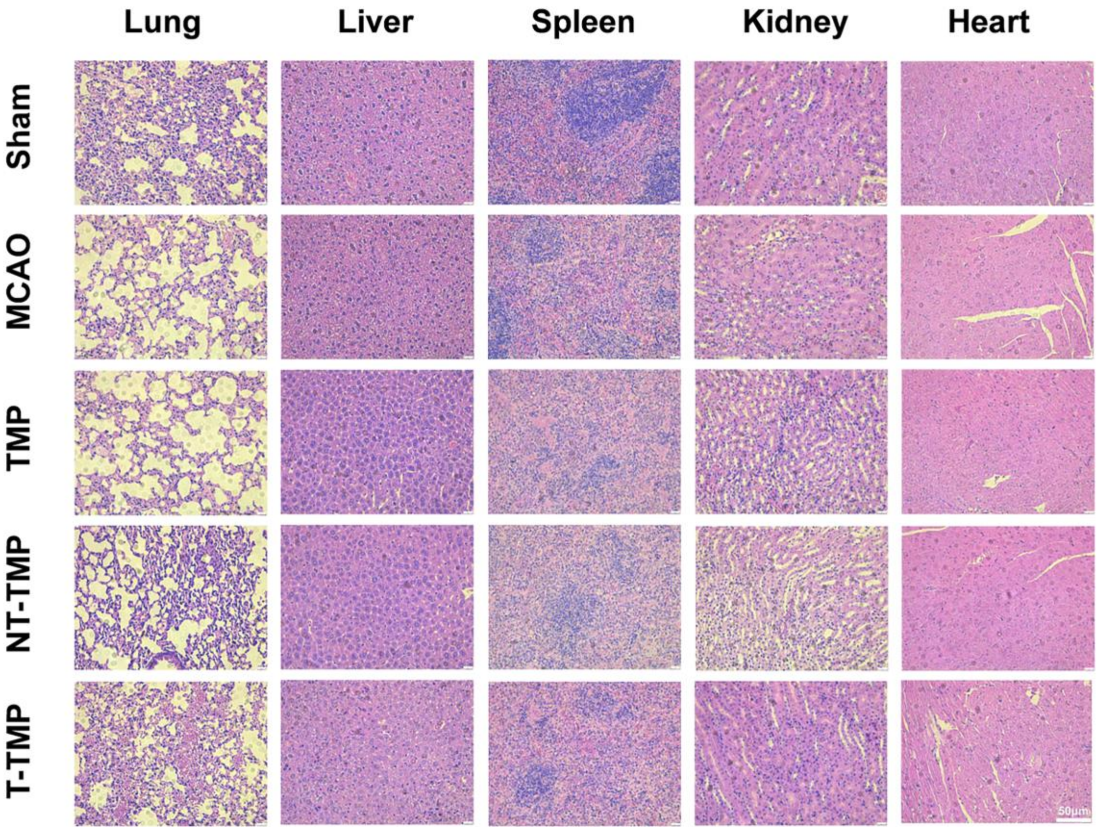

Figure S9. HE Staining of lung, liver, spleen, kidney, and heart showed no histology differences in Sham, MCAO, TMP, NT-TMP, and T-TMP groups.

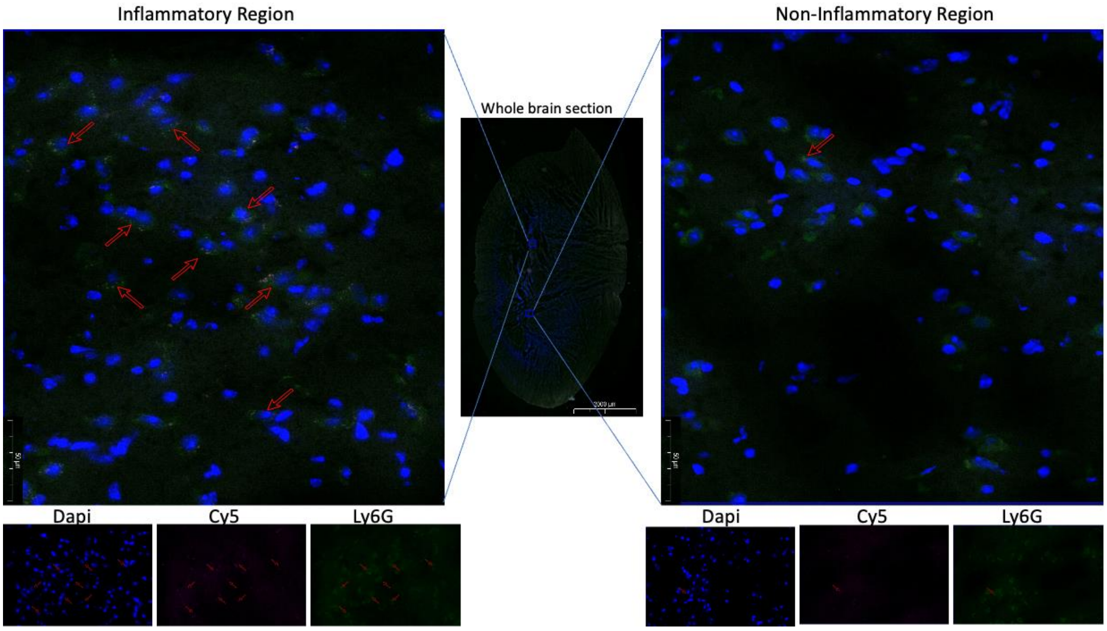

Figure S10. Representative image of whole brain sections from MCAO mice treated with T-TMP-Cy5 mouse. The zoomed windows in the image represent the inflammatory region and the normal region. Ly6G is stained with green fluorescence, while the pink fluorescence (dotted) of Cy5 represents the nanoparticles. Arrows are pointed at neutrophils with nanoparticle binding.
